# Nesfatin-1 Induces the Phosphorylation Levels of cAMP Response Element-Binding Protein for Intracellular Signaling in a Neural Cell Line

**DOI:** 10.1371/journal.pone.0050918

**Published:** 2012-12-06

**Authors:** Emi Ishida, Koshi Hashimoto, Hiroyuki Shimizu, Shuichi Okada, Tetsurou Satoh, Ikuo Kato, Masanobu Yamada, Masatomo Mori

**Affiliations:** 1 Department of Medicine and Molecular Science, Gunma University Graduate School of Medicine, Maebashi, Gunma, Japan; 2 Department of Health and Nutrition, Faculty of Health and Welfare, Takasaki University of Health and Welfare, Gunma, Japan; 3 Department of Bioorganic Chemistry, Faculty of Pharmaceutical Sciences, Hokuriku University, Kanazawa, Japan; University of Houston, United States of America

## Abstract

Nesfatin-1 is a novel anorexic peptide that reduces the food intake of rodents when administered either intraventricularly or intraperitoneally. However, the molecular mechanism of intracellular signaling via Nesfatin-1 is yet to be resolved. In the current study, we investigated the ability of different neuronal cell lines to respond to Nesfatin-1 and further elucidated the signal transduction pathway of Nesfatin-1. To achieve this, we transfected several cell lines with various combinations of reporter vectors containing different kinds of response elements and performed reporter assays with Nesfatin-1, its active midsegment encoding 30 amino acid residues (M30) and M30-derived mutants. Notably, we found that both Nesfatin-1 as well as M30, significantly increased cAMP response element (CRE) reporter activity in a mouse neuroblastoma cell line, NB41A3. An antagonist of Melanocortin 3/4 receptor, SHU9119, aborted the promoter activity, and a mutant M30, which exerts no anorexic effect *in vivo* did not induce the CRE reporter activity in NB41A3 cells. Western blotting analyses revealed that Nesfatin-1 and M30 significantly increased the phosphorylation levels of CRE-binding protein (CREB), without altering the intracellular cAMP levels. Further, our study showed that a mitogen-activated protein kinase (MAPK) kinase inhibitor and an L-type Calcium (Ca^2+^) channel blocker abolished the M30-induced CREB phosphorylation. Furthermore, the radio-receptor assay revealed that ^125^I-Nesfatin-1 binds in a saturable fashion to the membrane fractions of the mouse hypothalamus and NB41A3 cells, with Kd values of 0.79 nM and 0.17 nM, respectively. Collectively, our findings indicate the presence of a Nesfatin-1-specific receptor on the cell surface of NB41A3 cells and mouse hypothalamus. Our study highlights that Nesfatin-1, via its receptor, induces the phosphorylation of CREB, thus activating the intracellular signaling cascade in neurons.

## Introduction

Nesfatin-1, identified to be a novel satiety molecule is a hypothalamic neuropeptide derived from the precursor DNA binding/EF-hand/acidic protein (NEFA)/nucleobindin-2 (NUCB2). It has been demonstrated that Nesfatin-1 expression increased in the presence of troglitazone, an agonist of peroxisome proliferator-activated receptor-γ [1]. When we administered Nesfatin-1 intravenously or intraperitoneally to rats, their food intakes were decreased. However, when treated with SHU9119, an antagonist of Melanocortin 3/4 receptor (MC3/4R) in prior to administration of Nesfatin-1, we did not observe its anorexic effect. The thirty amino acid residues in the mid-segment of Nesfatin-1 (from 24 to 53 amino acid residues of Nesfatin-1, referred to M30) exert a comparable anorexic effect as full-length Nesfatin-1 [2], [3]. Recently, it has been demonstrated that Nesfatin-1 evoked calcium (Ca^2+^) influx into the neurons of the rat hypothalamus and induced insulin secretion from mouse pancreatic islets [4]–[7]. In addition, a study by Brailoiu et al. showed that Nesfatin-1 induces Ca^2+^ influx in mouse hypothalamic neurons, which was later found to be inhibited by the pertussis toxin [8]. Therefore, we speculated the existence of a Nesfatin-1 receptor on the mouse hypothalamus and that it could be a G protein-coupled receptor coupled to the Gi subunit. To our knowledge, to date, there have been no reports on Nesfatin-1 specific receptor and its role in the intracellular signaling cascade in neuronal cell lines. In view of the above, we demonstrated the existence of Nesfatin-1 receptor on the cell surface of a murine neuroblastoma cell line and mouse hypothalamus, and we further explored the role of Nesfatin-1 in the intracellular signal transduction pathway of neurons.

**Figure 1:**
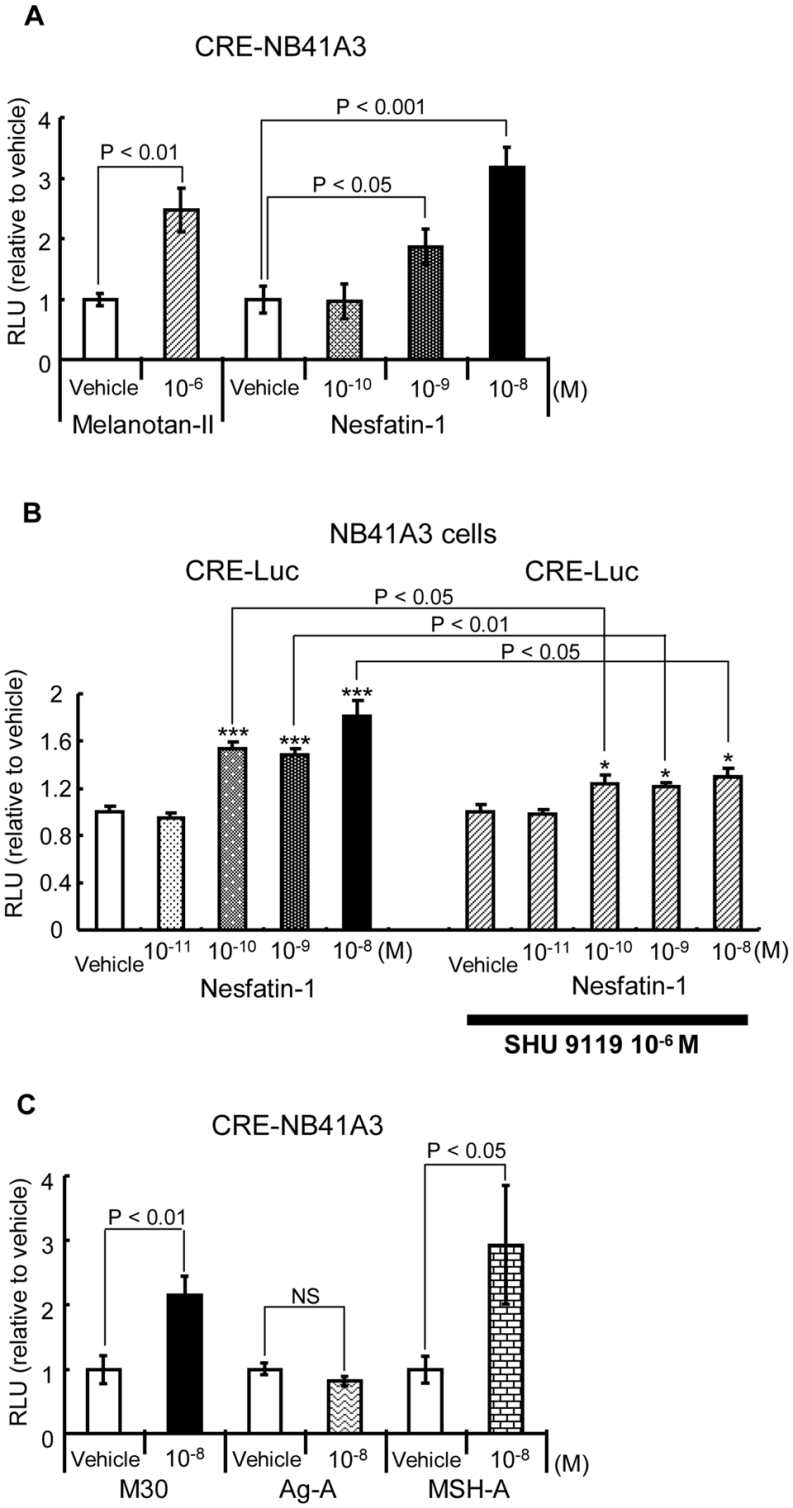
Nesfatin-1 induced CRE-Luc reporter activity in NB41A3 cells. A) Vehicles for Melanotan II and Nesfatin-1 were distilled water and 100 mM HEPES buffer (pH 7.5), respectively. Relative luciferase activities (mean ± SEM, n = 3) compared to the light units of CRE-Luc in the absence of Melanotan II or Nesfatin-1 (arbitrary light units divided by the total cellular protein and β-galactosidase [β-gal] activity) are shown as relative promoter activity (fold). The significant difference between the denoted pairs was obtained by the unpaired *t*-test. B) The antagonist of MC3/4R blocks the CRE reporter response by Nesfatin-1. Pretreatment of SHU9119 decreased the CRE-Luc reporter response induced by Nesfatin-1 in NB41A3 cells. Relative luciferase activities (mean ± SEM, n = 6) compared to the light units of CRE-Luc in the absence of Nesfatin-1 and SHU9119 (arbitrary light units divided by the total cellular protein and β-gal activity) are shown as relative promoter activity (fold). Asterisks show the significant differences between the promoter activity in the absence and presence of Nesfatin-1, in each panel. *** P<0.001, * P<0.05 by *t*-test. The significant differences between the absence and presence of SHU9119 in each concentration of Nesfatin-1 are denoted with lines. C) The midsegment of Nesfatin-1 (M30) but not a mutant M30 lacking the satiety effect *in vivo* induced the CRE-Luc reporter activity in NB41A3 cells. Relative luciferase activities (mean ± SEM, n = 3) compared to the light units of CRE-Luc in the absence of M30 or its derived mutants (arbitrary light units divided by cellular protein and β-gal activity) are shown as relative promoter activity (fold). The significant difference between the denoted pairs by *t*-test is shown. NS: not significant.

## Materials and Methods

### Cells

A mouse neuroblastoma (clone NB41A3) cell line was obtained from the American Type Culture Collection (ATCC No. CCL 147; Rockville, MD, USA) and employed for *in vitro* studies.

### Animals

For *in vivo* studies, 8- to 12-week-old male C57/BL6 (B6) mice were employed. All aspects of laboratory animal care were approved by the Institutional Animal Care and Use Committee of Gunma University Graduate School of Medicine (Maebashi, Gunma, Japan). Animals were maintained on a 12 h light/12 h dark schedule (lights on at 06:00 h) and fed laboratory chow as indicated and given water ad libitum.

**Figure 2:**
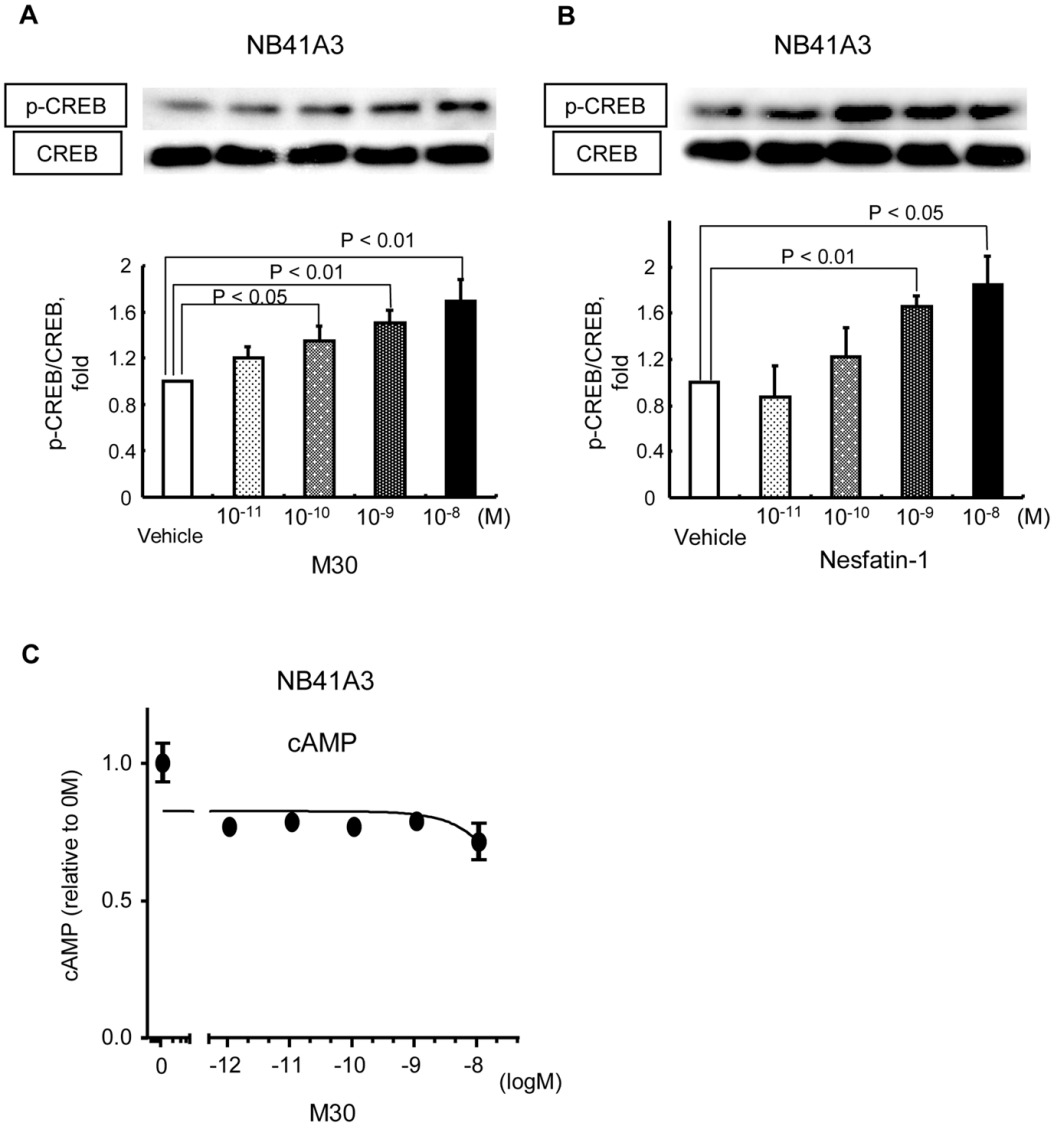
Nesfatin-1/M30 increased CREB phosphorylation, but not intracellular cAMP, levels in NB41A3 cells dose-dependently. A, B) Representative western blots for phospho-CREB (Ser 133) (pCREB) and CREB are shown in the upper panel. Relative optical densities of pCREB (mean ± SEM) normalized by CREB levels compared to the densities in the absence of M30 (A) or Nesfatin-1 (B) are shown as relative pCREB/CREB expression (fold). The significant difference between the denoted pairs by *t*-test is shown. C) Intracellular cAMP levels in NB41A3 cells were measured using an EIA kit. Relative cAMP concentrations (mean ± SEM) compared with the concentration in the absence of M30 are shown (fold). The concentrations of M30 added are shown as an exponential function with base 10.

### Reagents

^125^I-Nesfatin-1 was purchased from Phoenix pharmaceuticals (^125^I-Nesfatin-1 1–82) (Rat), #T-003-22, Burlingame, USA). The MC3/4R antagonist, SHU9119, was obtained from Bachem (Bubendorf, Switzerland, #1012060). Various drugs, namely, Melanotan II (Sigma-Aldrich, St. Louis, USA #M8693); Nimodipine (#482200, Merck KGaA, Darmstadt, Germany); KT5720 (#K3761, Sigma); Wortmannin (#W1628, Sigma); and PD98059 (#10006726, Cayman) were employed as chemical inhibitors of cell signaling pathway in the study.

**Figure 3:**
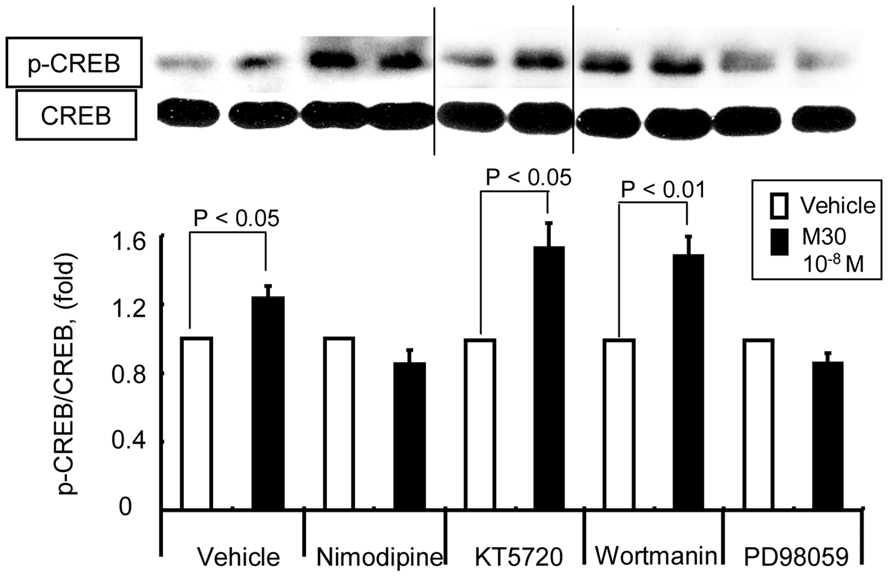
CREB phosphorylation by M30 is inhibited by L-type calcium channel blocker and MAPK kinase inhibitor. NB41A3 cells were pretreated with each inhibitor 30 min prior to Nesfatin-1 administration. Ethanol was used as the vehicle for nimodipine and DMSO was used for the others. Representative western blots for pCREB and CREB are shown in the upper panel. The data of vehicles shown in the figure is that of DMSO, and similar results were obtained in the presence of ethanol (data not shown). Relative optical densities of pCREB (mean ± SEM) normalized by CREB levels compared to the densities in the absence of M30 are shown as relative pCREB/CREB expression (fold).

### Nesfatin-1, M30, and its Derivative Mutant Peptides

Nesfatin-1 and M30 peptide were synthesized by Prof. Ikuo Kato (Hokuriku University, Ishikawa, Japan). The amino acid sequence of M30 is PDTGLYYDEYLKQVIEVLETDPHFREKLQK [2]. M30 mutant peptides (Ag-A and MSH-A) were synthesized at the Peptide Institute, Inc (Osaka, Japan). The amino acid sequences of Ag-A and MSH-A are PDTGLYYDEYAKAAAAALETDKHFREKLQK and PDTGLYYDEYLKQVIEVLETDAAAAEKLQK, respectively [2].

**Figure 4:**
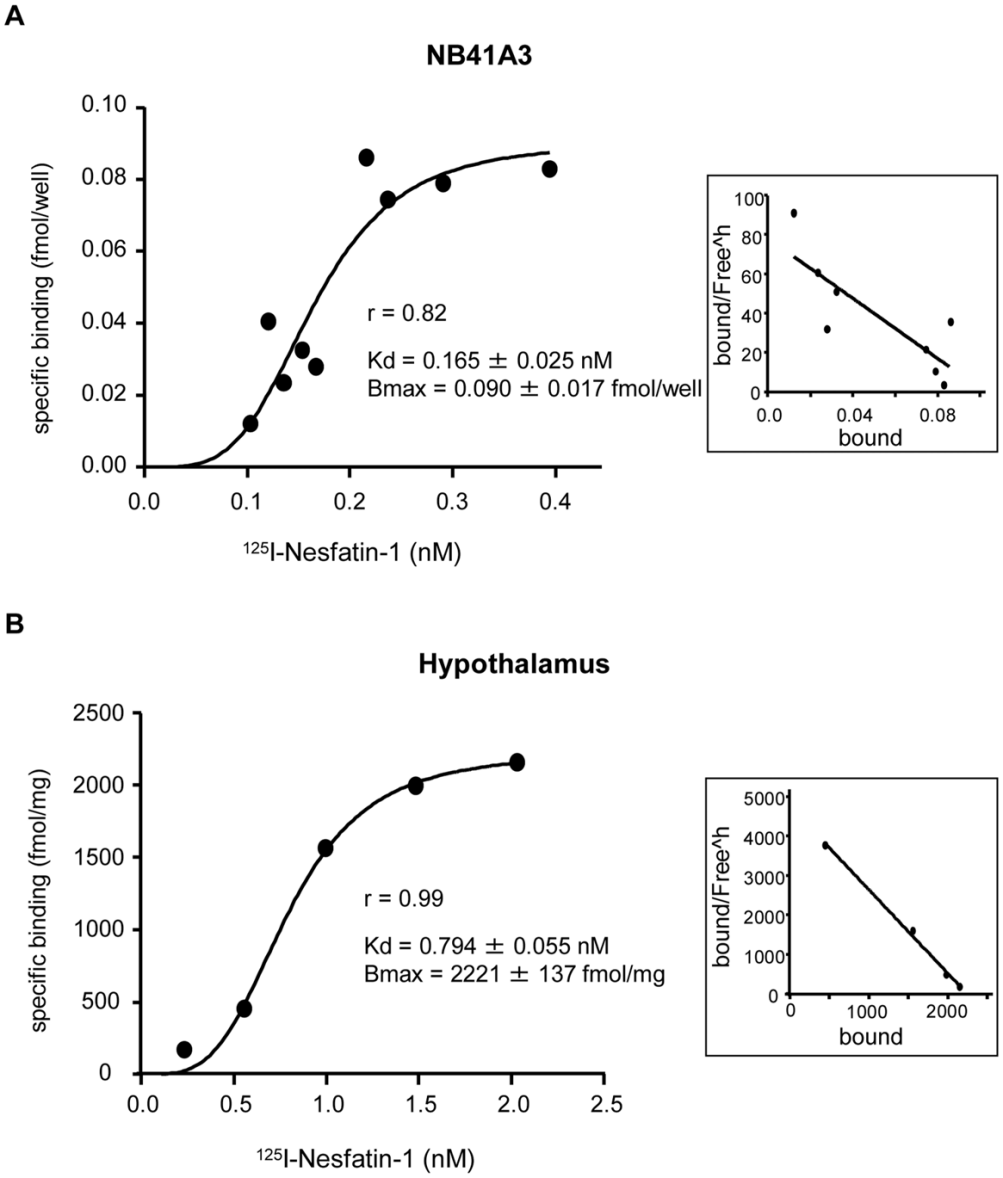
Nesfatin-1 specifically binds to the cell surface of NB41A3 cells and mouse hypothalamus. NB41A3 cells were plated in a 24-well format, and the total bound activity of ^125^I labeled Nesfatin-1 on collected NB41A3 cells was counted (A). The total bound activity of ^125^I labeled Nesfatin-1 on the membrane fraction of mouse hypothalamus was counted (B). Non specific binding was observed in the presence of excess of unlabeled Nesfatin-1. Saturation binding isotherm with Hill slope for specific binding after excluding nonspecific binding is shown. The Kd value and Bmax values were calculated by curve fitting program of Prism 5. Scatchard plot analysis is shown in the right panel.

### Establishment of Stable CRE-Luc-expressing Cell Lines

The cAMP response element (CRE)-Luc plasmid (pGL4.29[luc2p/CRE/Hygro]), with the luc2P synthetic firefly luciferase reporter gene under the control of the CRE promoter, was obtained from Promega. NB41A3 cells were transfected with the reporter vector, pGL4.29[*luc2P*/CRE/Hygro] vector using the lipofection method (Lipofectamine 2000, Invitrogen). Stably transfected NB41A3 cells expressing the CRE reporter vector was thus generated by transient transfection followed by hygromycin selection. Hereafter, these cell lines are referred to as “CRE-NB41A3.” To confirm the stable integration of CRE-Luc into NB41A3 cells, they were stimulated with Melanotan-II, a MC3/4R agonist and the CRE reporter activity was analyzed.

### Luciferase Assay

To measure the CRE reporter activity, the CRE-NB41A3 cells were plated on a 6-well plate for 72 h. Subsequently, the cultures were maintained in Dulbecco’s modified Eagle's medium with 10% double charcoal-stripped fetal bovine serum for 3 h either, in the presence or absence of Nesfatin-1, as described in the Figure legend. Data are presented as fold basal activation expressed as fold induction in the absence of ligand stimulation ± standard error of the mean (SEM) unless otherwise indicated. Luciferase activity is expressed as arbitrary light units per microgram of cellular protein. All the experiments were repeated at least twice with triplicate determinations.

### Western Blotting

In order to detect phosphorylated cAMP response element-binding protein (CREB), i.e., pCREB, western blot analysis was performed using rabbit monoclonal antibodies: anti-CREB (CREB (48H2) #9197, Cell Signaling Technology, Danvers, USA) and anti-Phospho-CREB (Ser133) (#9191, Cell Signaling Technology). NB41A3 cells were lysed in RIPA buffer containing phosphate-buffered saline (PBS), 1% Igepal CA-630 (Sigma), 0.5% sodium deoxycholate, 0.1% SDS, 1 mM dithiothreitol, 1 mM sodium orthovanadate, 2% (v/w) Complete™ protease inhibitor mixture (Roche Molecular Biochemicals), and 0.1 mg/mL phenylmethylsufonyl fluoride. Next, 25 µg of the whole cell extract was subjected to SDS-PAGE, and the intensities of the bands corresponding to CREB and pCREB were quantitatively measured by using the ImageJ software (Rasband, W.S., ImageJ, US National Institutes of Health, Bethesda, Maryland, USA, http://imagej.nih.gov/ij/, 1997–2011). The images were processed and analyzed using Adobe Photoshop 7.0 (Adobe Systems Corp., San Jose, CA). All experiments were independently repeated at least thrice, and similar results were thus obtained. Levels of pCREB protein levels were normalized to that of total CREB. Data are presented as fold basal activation expressed as fold induction in the absence of ligand stimulation ± SEM unless otherwise as indicated.

### Enzyme Immunoassay (EIA) for Intracellular Cyclic AMP

Levels of intracellular cyclic AMP were determined by enzyme immunoassay (EIA) using the cyclic AMP EIA kit (#581001, Cayman, Ann Arbor, USA). cAMP EIA was performed according to the manufacturer’s instructions. Briefly, NB41A3 cells were seeded in 6-well tissue culture plates and cultured for 48 h in Dulbecco’s modified Eagle’s medium supplemented with 10% double charcoal-stripped fetal bovine serum. Subsequently, ligands were added to stimulate the cells. After 7 min of incubation with the ligands, the medium was changed to 0.1 M HCl, and the cells were further incubated for 20 min before cell lysis. The cell suspensions were collected, and the lysates were centrifuged at 1,000×*g* for 10 min at room temperature. The samples were then acetylated using the reagents provided by manufacturer. The protein content in the supernatants was determined by Bradford protein assay. Each sample was analyzed in triplicate, and the analysis was repeated more than 3 times with similar results. The values represent mean ± SD of triplicates.

### Receptor Binding Assays

To confirm the presence of Nesfatin-1 specific receptor on the cell surface of NB41A3 cells and the cell membrane fraction of mouse hypothalamus (HPT), we performed radio-receptor assays by using ^125^I-Nesfatin-1 and unlabeled Nesfatin-1. The ^125^I-Nesfatin-1 specific activity was 1,800 Ci/mmol. NB41A3 cells were cultured in a 24-well format. Approximately 80–90% confluent cells were washed with PBS, treated with various concentrations of ^125^I-Nesfatin-1, and incubated at room temperature for 2 h. In order to observe nonspecific binding of NB41A3 cells, cold Nesfatin-1 (minimum 2,500-fold) at an absolute concentration of 1 µM was added. After the cells were washed twice with PBS, they were dissolved in 0.1 N NaOH, and then collected using cotton swabs. The radioactivity in the cotton swabs was measured by a gamma-ray counter. To determine whether Nesfatin-1 binds to the cell surface of mouse HPT, HPT was excised from wild-type C57/B6 male mouse (8–12 weeks old) and homogenized in 10 volumes of 40 mM Tris-HCl and sucrose by using the Polytron homogenizer. The fractionation was done by ultracentrifugation (1,000 g for 10 min then 56,000 g for 30 min). To obtain the membrane fraction, the pellet of cell debris obtained after cell sonication and centrifugation was resuspended in 40 mM Tris-HCl with 0.2% bovine serum albumin (BSA). The membrane fraction was incubated with ^125^I-Nesfatin-1 in the presence or absence of cold Nesfatin-1 (5,000-fold at an absolute concentration of 10 µM) overnight at 4°C. The reaction mixture was filtered through a BSA-treated GF/B glass-fiber filter (Millipore). Subsequently, the filter was washed 4 times with 40 mM Tris-HCl and BSA, and the bound radioactivity was measured using a gamma-ray counter.

### Statistical Analyses

Statistical analyses were performed using GraphPad Prism 5 (GraphPad Software, Inc., La Jolla, CA). Values are expressed as the mean ± SEM. The significance of differences between the mean values was evaluated using the unpaired Student’s *t*-test followed by one-way analysis of variance (ANOVA).

## Results

### Nesfatin-1 Activates CRE Reporter in NB41A3 Cells

In order to determine the responsiveness of several cell lines to Nesfatin-1, we introduced reporter vectors containing various kinds of response elements into them by transient transfection and performed reporter assays with Nesfatin-1 (data not shown). Among numerous combinations of cell lines and reporters, we identified that Nesfatin-1 activated CRE-Luc reporter in NB41A3 cells, derived from a mouse neuroblastoma cell line. On the basis of this preliminary data, we established stable NB41A3 cell lines expressing CRE-Luc by selecting cells resistant to hygromycin. As shown in Figure 1A (left graphs), Melanotan-II, an agonist of MC3/4R, significantly induced the reporter activity, indicating that CRE-Luc was successfully integrated into the cells. In addition, Nesfatin-1 significantly activated the luciferase activity in a dose-dependent manner (Figure 1A, right graphs). On the other hand, SHU9119, which is antagonist of MC3/4R, attenuated the reporter activity induced by Nesfatin-1 (Figure 1B). Further, it was observed that NB41A3 cells expressed MC4R both at the mRNA and protein levels (data not shown).

### M30 also Induces CRE Reporter in NB41A3 Cells and the AgRP-similar Amino Acid Sequence in M30 is Essential for the Effect

We have previously demonstrated that M30, synthesized from 24 to 53 amino acid residues of Nesfatin-1, exerted a comparable satiety effect *in vivo* [2], [3]. We also synthesized 2 different M30 mutants [2], [3]. In the first mutant, the α-MSH-similar amino acid sequence was substituted with alanine residues; this mutant was designated as M30 MSH-A. In the second mutant, the AgRP-similar amino acid sequence was substituted with alanine residues; this mutant was designated as M30 Ag-A. In our earlier studies, we had noted that MSH-A mutant, but not that of Ag-A, exerted an anorexic effect *in vivo* [2], [3]. As shown in Figure 1C, unlike the Ag-A mutant, MSH-A as well as the wild-type M30 significantly induced the CRE-Luc activity in CRE-NB41A3 cells. This data indicated that anorexic response was induced by M30 via the CRE signaling pathway.

### Nesfatin-1/M30 Induce CREB Phosphorylation but not Intracellular cAMP Levels in NB41A3 Cells

Western blotting analysis revealed that pCREB levels in NB41A3 cells significantly increased in an M30 dose-dependent manner (Figure 2A). Similar results were obtained with Nesfatin-1 (Figure 2B). However, the intracellular cAMP levels in NB41A3 cells were not affected by M30 peptide administration (Figure 2C).

### M30 Induces CREB Phosphorylation in NB41A3 Cells through Ca^2+^ Channels and MAP Kinase Pathway

To elucidate the molecular mechanism underlying CREB phosphorylation by Nesfatin-1/M30, we performed western blot by employing several inhibitors of cell signaling. Notably, the phosphorylation of CREB was abolished in the presence of nimodipine, a type-L Ca^2+^ channel blocker, and PD98059, a mitogen-activated protein kinase (MAPK) kinase inhibitor (Figure 3). Therefore, our findings suggest that the increase in pCREB levels induced by M30 requires Ca^2+^ channels and the MAPK pathway.

### Nesfatin-1-specific Receptor Exists on the Cell Surface of NB41A3 Cells and the Cell Membrane Fraction of Mouse HPT

We performed radio-receptor assays with NB41A3 cells and the cell membrane fraction of mouse HPT and investigated the specificity of ^125^I-Nesfatin-1 binding. It was observed that the percentage of nonspecific binding was 80–90% in the cell plate-based assay and 50–60% in glass filter-based assay. Our results demonstrated a specific binding of Nesfatin-1 to the cell surface of NB41A3 cells as well as the cell membrane fraction, where the Kd values were 0.165±0.025 nM and 0.794±0.055 nM, respectively (Figure 4A, B). These findings suggest that Nesfatin-1 acts via a specific cell surface receptor in its action on NB41A3 cells as well as the cell membrane fraction of mouse hypothalamus.

## Discussion

The present study is the first of its kind to demonstrate that Nesfatin-1 induces CRE reporter activity in NB41A3 cells and acts via a specific cell surface receptor to induce the phosphorylation of CREB, thereby activating the intracellular signaling cascade in neuronal cells. Thus far, the only established neuronal cell line that responds to Nesfatin-1 is the murine neuroblastoma cell line (NB41A3).

In our study, MC4R was shown to be expressed on NB41A3 cell surface and Melanotan-II, which is an MC4R agonist, activated CRE-Luc reporter in the cells. It has been established that the MC4R signaling pathway is essential to the anorexic effect evoked by Nesfatin-1 *in vivo* [1]. Corroborating the *in vivo* data, the results of our study demonstrated the attenuation of Nesfatin-1-induced CRE reporter activity by SHU9119, an antagonist of MC4R. Further, these findings led to the hypothesis that Nesfatin-1 utilizes the CRE signaling pathway for its satiety effect *in vivo*. Strengthening our hypothesis, several earlier studies reported the involvement of CRE in the central regulation of feeding behavior and energy expenditure [9], [10].

In the present study, we demonstrated that the M30 peptide also activated CRE reporter in addition to Nesfatin-1 in NB41A3 cells (Figure 1C), indicating that M30 shares a similar intracellular pathway with Nesfatin-1 in order to exert the anorexic effect. Our findings are in agreement with those of our previous studies, which showed that the M30 synthesized from 24 to 53 amino acid residues of Nesfatin-1 exerted a comparable satiety effect *in vivo* [2], [3]. Moreover, in our previous reports, we demonstrated that the midsegment region of Nesfatin-1 was responsible for inducing anorexia *in vivo* and showed a high similarity to AgRP rather than alpha-MSH [2], [3]. In the current study, the M30 derived Ag-A mutant, wherein the AgRP-similar amino acid sequence of the M30 mutant was substituted with alanine residues, did not activate CRE reporter in NB41A3 cells, indicating that the AgRP-similar amino acid sequence in M30 mutant plays a crucial role in the intracellular signaling pathway evoked by M30.

Despite the increase in the phosphorylated CREB levels in NB41A3 cells, which was induced by M30, the levels of intracellular cAMP concentration did not increase. Furthermore, a specific protein kinase A blocker, KT5720, did not affect M30-induced CREB phosphorylation in NB41A3 cells, indicating that M30 does not act through the protein kinase A pathway.

Brailoiu et al. hypothesized that Nesfatin-1 specific receptor may be a G protein-coupled receptor and that Gi/o protein might be involved, as the Ca^2+^ influx into the cultured rat hypothalamic neurons was found to be inhibited by pertussis toxin [8]. Strengthening this hypothesis, the results of the current study demonstrated that the L-type Ca^2+^ channel blocker, nimodipine, and the MAPK kinase inhibitor, PD98059, blocked the M30-induced CREB phosphorylation, thus suggesting that Ca^2+^ influx and/or MAPK signaling pathway activates pCREB. Taken together, our results therefore confirm that Nesfatin-1/M30 specific receptor may be a Gi/o protein-coupled receptor and that Nesfatin-1/M30 utilizes Ca^2+^ influx and/or the MAPK signaling pathway to phosphorylate CREB in NB41A3 cells.

However, our results slightly differ from those of a study by Brailoiu et al., which showed that a specific protein kinase A blocker, KT5720, inhibited Ca^2+^ influx into the cultured rat hypothalamic neurons [8]. This discrepancy could be attributed to the stark differences between the cultured rat hypothalamic neurons and the NB41A3 cell lines.

We have, for the first time, demonstrated that Nesfatin-1 specific receptor exists on the cell surface of NB41A3 cells and mouse HPT. The Kd values obtained from both NB41A3 cells and mouse HPT are in the 10^−10 ^M order. This finding is in agreement with previous studies, which reported a similar value for EC50 [4]–[8]. However, the Kd value of NB41A3 cells was smaller than that of mouse HPT. Even though it cannot be confirmed whether the difference is significant, we speculate that it could be due to the difference between intact cell binding assays and assays with isolated membrane preparations. The nonspecific binding in the whole cells was extremely high compared to that in the isolated membranes. We presume that this could be due, among other things, to internalization of the radiolabelled ligand, which can be observed in intact cell assays and that the Kd value obtained with the membranes should be closest to the ‘true’ one [11].

Our study highlights the utility of stable CRE-Luc-transfected NB41A3 cells in identifying the Nesfatin-1 specific receptor. Exciting preliminary data from recent studies revealed that Nesfatin-1 exerts pleiotropic actions and suggests its potential role in intracellular signaling machinery [12],[13]. Therefore, future studies investigating the functional role of Nesfatin-1 in intracellular signaling cascades and focusing on the identification of the Nesfatin-1 specific receptor are warranted. Furthermore, NB41A3 cells and CRE-NB41A3 stable cell lines could be employed to validate the synthesized Nesfatin-1 and M30 as well as its derived peptides.

## References

[1] Oh-I S, Shimizu H, Satoh T, Okada S, Adachi S, et al (2006) Identification of nesfatin-1 as a satiety molecule in the hypothalamus. Nature 443: 709–712. 17036007 10.1038/nature05162

[2] Shimizu H, Oh-I S, Hashimoto K, Nakata M, Yamamoto S, et al (2009) Peripheral administration of nesfatin-1 reduces food intake in mice: the leptin-independent mechanism. Endocrinology 150: 662–671. 19176321 10.1210/en.2008-0598

[3] Shimizu H, Ohsaki A, Oh-I S, Okada S, Mori M (2009) A new anorexigenic protein, nesfatin-1. Peptides 30: 995–998. 19452636 10.1016/j.peptides.2009.01.002

[4] Iwasaki Y, Nakabayashi H, Kakei M, Shimizu H, Mori M, et al (2009) Nesfatin-1 evokes Ca2+ signaling in isolated vagal afferent neurons via Ca2+ influx through N-type channels. Biochemical and biophysical research communications 390: 958–962. 19852938 10.1016/j.bbrc.2009.10.085

[5] Nakata M, Manaka K, Yamamoto S, Mori M, Yada T (2011) Nesfatin-1 enhances glucose-induced insulin secretion by promoting Ca^2+^ influx through L-type channels in mouse islet beta-cells. Endocrine journal 58: 305–313. 21325742 10.1507/endocrj.k11e-056

[6] Gonzalez R, Reingold BK, Gao X, Gaidhu MP, Tsushima RG, et al (2011) Nesfatin-1 exerts a direct, glucose-dependent insulinotropic action on mouse islet beta- and MIN6 cells. The journal of endocrinology 208: R9–R16. 21224288 10.1530/JOE-10-0492

[7] Gonzalez R, Perry RL, Gao X, Gaidhu MP, Tsushima RG, et al (2011) Nutrient responsive nesfatin-1 regulates energy balance and induces glucose-stimulated insulin secretion in rats. Endocrinology 152: 3628–3637. 21828181 10.1210/en.2010-1471

[8] Brailoiu GC, Dun SL, Brailoiu E, Inan S, Yang J, et al (2007) Nesfatin-1: distribution and interaction with a G protein-coupled receptor in the rat brain. Endocrinology 148: 5088–5094. 17627999 10.1210/en.2007-0701

[9] Shimizu-Albergine M, Ippolito DL, Beavo JA (2001) Downregulation of fasting-induced cAMP response element-mediated gene induction by leptin in neuropeptide Y neurons of the arcuate nucleus. J Neurosci. 21: 1238–1246. 10.1523/JNEUROSCI.21-04-01238.2001 PMC6762228 11160394

[10] Sheriff S, Chance WT, Fischer JE, Balasubramaniam A (1997) Neuropeptide Y treatment and food deprivation increase cyclic AMP response element-binding in rat hypothalamus. Mol Pharmacol. 51: 597–604. 10.1124/mol.51.4.597 9106624

[11] Bylund DB, Toews ML (1993) Radioligand binding methods: practical guide and tips. Am J Physiol. 265: L421–L429. 10.1152/ajplung.1993.265.5.L421 8238529

[12] Shimizu H, Oh-I S, Okada S, Mori M (2009) Nesfatin-1: an overview and future clinical application. Endocrine journal 56: 537–543. 19461159 10.1507/endocrj.k09e-117

[13] Stengel A, Tache Y (2011) Minireview: nesfatin-1-an emerging new player in the brain-gut, endocrine, and metabolic axis. Endocrinology 152: 4033–4038. 21862618 10.1210/en.2011-1500 PMC3199002

